# Monthly Summary

**Published:** 1892-02

**Authors:** 


### Monthly Summary.

TUMENOL.—While the list of new remedies that are constantly being brought to the profession's notice is very large, and the number of valuables ones comparatively small, yet, in spite of a gradually increasing feeling of caution on the part of the practitioner in embracing these newer preparations, those which possess any real merit are usually soon recognized. Thus it is that tumenol, a substance obtained from mineral oils, and recently introduced by the dermatologist,

Dr. Neisser, appears to have come to stay, and is certainly daily gaining ground among these specialists abroad. The drug is used only as a topical application, and in the form of tincture, solution, oil, salve, or plaster; and, broadly speaking, occupies a position similar to that now held by zinc, with the exception that the claims for tumenol are even greater than those of the former useful substance, at least, in dermatological practice.

The solution has been proven to act more beneficially in cases of acute recurring eczemas of the face and hands, and also in both acute and chronic eczemas of the extremities. In such cases it is used in a two or five per cent. solution in the form of a wet dressing. It forms a dry protective skin, and greatly allays the itching.

Neisser has also used the solution and tumenol gauze in the treatment of purulent and other discharges from the vagina; but while the remedy was well tolerated, the curative effects obtained were but slight.

Its greatest usefulness is undoubtedly when used as a salve or ointment of the strength of five or ten per cent. It may be made with vaseline or anhydrous lanolin as a base, and of a varying consistency subject to the requirements of he case. Again, the drug may be added to zinc ointment.

Various writers have repeatedly shown how tumenol ointment may be used with greater efficiency in almost every case in which zinc ointment is now used, and its supporters claim that its superiority over the latter preparation is most marked.

Upon ulcerating surfaces in cases of lupus, contagious impetigo, pemphigus, and the periods of desquamation and exfoliation of various other skin diseases, tumenol ointment is claimed to have been used with signal success.

In dry and squamous periods of various skin diseases the tincture gives better results than the salve. The tincture yields excellent results in almost any erosion accompanied by itching.

Tumenol-soap plaster seems to act better and more rapidly than either the plain or salicylated plaster. The oil when applied to the vesicles of eczematous patches acts promptly and beneficially.

Neisser has also used finely powdered tumenol-sulphonic acid as a dusting powder on ulcerating surfaces with excellent results.

In conclusion, the most recent reports of the drug justify our recommending it as a quick drier, a reducer of inflammation, and a healing and soothing application in eczemas, erosions, excoriations, superficial ulcerations, and various forms of pruritus. Its action upon the infiltrations of eczema is but slight, its beneficial influence being confined to the superficial, catarrhal, or desquamative forms of the disease, and especially in the acute forms.

The drug is undoubtedly a valuable one and deserving of serious consideration and trial.—*Med. and Surg. Reporter.*

---

CHROMIC ACID IN SYPHILITIC AFFECTIONS OF THE MOUTH.—For a long time past various largyngologists have recognized in chronic acid a valuable remedy for many diseases of the mouth and pharynx. Its special value is in syphilitic affections of this region, and within a recent date many writers of note have sought to bring the subject before the profession through the medium of medical journals. Among the most recent writings on the subject we have before us those from the pens of Schuster, Vidal, Paget and Feibel.

In placing the results of their experiences before our readers, we merely hope to give them points regarding the most judicious methods for the application of the drug, laying no claim for the introduction of any new therapeutic means, since, without doubt, chronic acid has long been recognized as one of our "stand-byes" in the treatment of syphilitic affections of the mouth.

Schuster has made it a practice first to scarify the syphilitic patches, and then apply a concentrated solution of the acid to the parts. This procedure he repeats once or twice a week until a cure is obtained. His results have been most gratifying.

Vidal, in similar affections uses a caustic solution of the acid (1 to 8, or equal parts of water). This he applies locally for one or two minutes, and then washes the parts with clean water. The treatment is repeated every three or four days until a cure is obtained.

Paget's contribution to the subject consists mainly in a record of the remarkable success which has attended the use of the acid in cases of lingual psoriasis.

Bethin, the London laryngologist, recommends the local application of the acid in cases of chronic superficial glossitis occurring in subjects who have previously had syphilis, and found mostly among habitual drinkers. Another writer, Butlin, recommends the local application of chronic acid three times a day, in the form of a half per cent. solution, as being most valuable in cases of ulcers of the tongue.

Finally, Dr. Ernest Feibel has used the acid in a variety of syphilitic affections of the mouth with such success that he has discarded all other remedies for it. He uses invariably a cauterizing solution of one part of the acid to two parts of water. In cases of syphilitic ulcers of the tongue when a long continued internal treatment had yielded no results, a few applications of the acid produced a complete disappearance of the sores. In these cases the remedy should be applied about every second day, and a week will usually suffice to bring about a cure. In cases of indurated lumps on the tongue a very few applications will effect a cure. The "opaline plagues" also disappeared quickly under its use, as well as ulcers in the angles of the mouth.

In that most troublesome and stubborn form of oral syphilis, described as *lingua geographica*, no other remedy acts in so potent a manner as chromic acid. In such cases the tongue should be first dried, then the solution applied with a camel's-hair brush, and after a few minutes the parts carefully washed. The cauterized tissue will be thrown off in the course of three or four days, and the normal tissue will be found under it. Four or five applications during the course of about two weeks will usually effect a cure. Naturally, cases have been reported in which the remedy did not give entire satisfaction. But in the large majority chromic acid will be found of inestimable value in syphilitic affections of the mouth, bringing about an immediate beneficial action, and leaving as a result a healthy condition of the mucous membrane.—*Med. and Surg. Reporter.*

---

A NEW STYPTIC.—The styptics ordinarily employed in medicine and surgery act by causing contraction of the blood-vessels, by increasing the coagulability of the blood, or lowering the action of the heart. While styptics fulfil the required conditions fairly well when locally applied, they are very uncertain agents when given internally. So far as increasing the coagulability of the blood is concerned, such substances as lead, gallic acid, and the ferric salts are of little value.

We read with some interest, therefore, the description given by Dr A. E. Wright, in the *British Medical Journal* of December 19, 1891, of a new styptic which he asserts does undoubtedly increase the coagulability of the blood, both when used externally and internally. Dr. Wright's remedy consists of two substances, viz., the physiological ferment of the blood and certain salts of time.

The fibrin ferment is obtained from the blood of herbivora, preferably the sheep, in the following way: The blood is received from the vessels into three times its volume of ordinary water. It is then set aside for a few minutes until it commences to gelatinize, when it is stirred in the ordinary manner with a bundle of sticks or of wires. The fibrin that is collected in this manner from the diluted blood is composed of very much finer threads than that prepared from the undiluted blood, and it also presents the advantage of enclosing much fewer blood-corpuscles. The fibrin thus obtained is washed out under a tap, or, better, in several changes of ordinary water until it is practically free from blood-pigment. This process of washing can usually be efficiently completed in the course of some ten minutes, and since the fibrin ferment is soluble in water it is expedient not to overdo the washing. The fibrin is now ready for use, and the fibrin ferment is to be obtained from it by extraction with a moderate quantity of water—approximately some five to ten volumes of water may be used to each portion of fibrin. The extraction is to be continued for some hours (I have usually extracted for twentyfour hours). The filtered extract constitutes fibrin ferment solution. To this is added one per cent. of calcium chloride.

Instead of proceeding immediately to the extraction of the fibrin ferment it is preferable to keep the fibrin ferment for a few days at least under strong alcohol (it may be left there practically indefinitely without loss of its property of yielding fibrin ferment). In such cases the alcohol must naturally be removed by either washing or pressing between filter-paper before proceeding to the extraction. If the extraction is now made with distilled water that has been boiled before using, there is evidently no difficulty in obtaining an aseptic fluid by this method.

The calcium chloride is added because it has been shown that its presence increases the coagulability of the blood. The mixture is to be applied locally. Its hypodermic injection in man is suggested but has not been tried.

Dr. Wright dwells more particularly upon the probable value of the internal use of calcium chloride alone. From experiments on dogs he shows that it apparently increases the coagulability of the blood. He suggests its use, therefore, in internal hemorrhages and in hæmophilia and aneurisms. The dose for a man is not given, but we infer that it must be large, *i. e.*, one or two drachms. It is to be regretted that Dr. Wright did not have the patience to make mere practical tests of his styptic substances. They at least seem to be rational and safe.—*Med. Record.*

---

PHENOCOLL HYDROCHLORATE.—We glean the following from a communication by Dr. Isaac Ott, in the *Journal Nervous and Mental Diseases*, Feb., 1892:

This body is a white crystalline powder, soluble in sixteen parts of water at 62° F. It is the hydrochlorate salt of amido-acet-para-phenetidin. This new combination is a phenacetin rendered soluble by the addition of an amido group. Alkalies and alkaline carbonates throw down from the hydrochlorate solution the pure base phenocoll. The pure base is soluble in hot water, but dissolves to only a small extent in cold water.

In experiments on rabbits, Dr. Ott learned that: The action of phenocoll upon the circulation is one of depression. The pulse and arterial pressure fall after a dose by the jugular. The reduction of the pulse is not due to an excitation of the pneumogastric, but the cause must reside in the heart itself. It is reasonable to infer that weakness of the heart itself is one of the principal factors in the depression of arterial tension. Phenocoll first increases the respiratory movements and then reduces them. Previous division of the vagi does not change the state of affairs. It temporarily reduces the temperature of the body.

Phenocoll is a drug which may be recommended in the pains of influenza, rheumatism and gout. In the neuralgias of the intercostal or other peripheral nerves it will be found valuable. As an antipyretic its action is not of long endurance. Unlike phenacetin, it is soluble in water and it is probably quite as effective for the subjugation of pain. For the reduction of fever phenacetin is superior.—*Md. Medical Journal.*

---

ENLARGEMENT OF THE CERVICAL GLANDS, DUE TO THE IMPACTION OF LOWER LEFT WISDOM TOOTH.—Mr. A., age 21, a strong, healthy young man, without any tubercular history, consulted Dr.—— regarding an enlargement of the cervical glands. Dr.——, apprehending dental irritation as the cause, advised him to see a dentist. This he accordingly did consulting me on the 8th of the present month. On examination, I found the glands considerably enlarged, painful on pressure, also pain at the angle of the lower jaw. In the mouth, the teeth in the vicinity were found well developed, sound and healthy. The third molar was, however, unerupted, the point of the anterior cusp being visible at the line of the neck of the second molar. On applying pressure to the surrounding parts, pus could be evacuated, having its exit at the erupted portion of the tooth. This symptom, connected with the evident want of space for the reception of the tooth, led me to advise its removal. This the patient acceded to after, on my advice, consulting his medical attendant. The operation of removal was one of extreme difficulty, a considerable degree of resistance being experienced, but it was eventually dislodged with a Coleman's straight elevator. The tooth proved to be a large, well developed three rooted molar, measuring from crown to apex fully seven eights of an inch. The patient was seen the following day. The jaw was slightly stiffened, and the glandular pain intensified, which was to be expected, being due to the force employed in the removal of the tooth. Three days thereafter, there was a marked diminution of pain and swelling, the general characteristics were favorable, and pointed to a satisfactory and complete cessation of all existing complications.—*London Dental Record.*

---

A CASE IN PRACTICE.—By R. E. Sparks, Kingston, Ont.—A gentleman called upon me about two years ago, suffering from sore throat on the left side, neuralgic pains in ear and side of face and head. His physician had been unable to treat the trouble successfully, or even to diagnose the cause. I found, upon examination, that he was biting his cheek severely between his wisdom teeth. I removed the upper one and dismissed him. A few days since he returned to my office and reported having derived immediate relief after the operation.

This time he had come to have another tooth extracted, as he was biting his cheek opposite his first molars upon the same side. Upon examination I found a tumor, about as large as a good sized pea, upon the inside of the cheek, opposite the teeth mentioned. His teeth were sound but worn off flat. The outer edges were quite sharp. His cheeks were fleshy, and as soon as he opened his mouth the tumor passed in between the teeth, and lay in position to be bitten when his teeth came together. I ground off the sharp edges of the teeth, grasped the tumor with a pair of tongue forceps and drew it gently into the mouth, while my assistant held the cheek in position with a mouth mirror. The tension upon the tumor made it an easy matter to snip it off at its base with a pair of curved scissors. A jet of cold water thrown into the wound from an ordinary dental syringe, for a few minutes, stopped the slight bleeding which had taken place. The patient left delighted that he had been relieved of his trouble without the loss of his grinder.—*Dominion Dental Journal.*

---

ULCERS AND GANGRENOUS WOUNDS.—Dr. Brown (*Lo Spcrimentale*, No. 16. 1891) uses with success in ulcers and gangrenous wounds the following solution:

| | | | |
|---|---|---|---|
| R | Zinc sulphat | - - - | grammes 5.75. |
| | Aq. destillat | - - | " 600. |
| | Acid sulph. dil | - - | " 16. |

The parts are first washed with warm water and then the antiseptic solution applied on cotton. This dressing is changed every three hours. This causes the fœtid odor to disappear and promotes cicatrization—*Med. and Surg. Reporter.*

---

A CONVENIENT METHOD OF ADDING NEW TEETH TO OLD PLATES.—By R. E. Sparks, Kingston, Ont.—Dry the plate and stick on a piece of soft wax opposite where each tooth is to be added. Replace the plate in the mouth. If the case be one where the teeth to be added are to replace some which have been extracted, press the soft wax up over the gum. This gives you an impression of the part with the plate in place. While the wax is still soft have the patient close the mouth. This gives you an articulation opposite where the teeth are to be added. While the mouth is shut, see that the wax is not forced away from the gum by the occlusion. Then with a pledget of cotton, dipped in cold water, the wax can be hardened in a moment. You may now dismiss the patient. Remove the plate and run cast. As soon as hard, turn over, and run a little plaster in the articulation, letting it extend to a couple of the teeth on the plate. When this is hard, lift off, and remove the wax. The teeth may now be ground and articulated. This method saves much time for patient and operator and ensures accuracy and may all be done by the laboratory assistant except the taking of the impression and articulation.—*Dominion Dent. Journal.*

